# Genetic associations of plasma biomarkers of neurodegeneration in a population of laboratory marmosets

**DOI:** 10.1002/alz70855_103724

**Published:** 2025-12-23

**Authors:** Sonal Kumar, Annat Haber, Catrina Spruce, Laura Schultz, Hasi Huhe, Takeshi Murai, Sang‐Ho Choi, Stephanie Hachem, Yongshan Mou, Seung‐Kwon Ha, Jung Eun Park, Lauren Bailey, Lauren Mongeau, Andrew DeSana, Brianne Stein, Lauren K Hayrynen Schaeffer, Gregg E Homanics, Afonso C Silva, Stacey J Sukoff Rizzo, Gregory W Carter

**Affiliations:** ^1^ Tufts University Graduate School of Biomedical Sciences, Boston, MA, USA; ^2^ The Jackson Laboratory, Bar Harbor, ME, USA; ^3^ The Jackson Laboratory for Genomic Medicine, Farmington, CT, USA; ^4^ University of Pittsburgh School of Medicine, Pittsburgh, PA, USA

## Abstract

**Background:**

Alzheimer's disease (AD) is a progressively neurodegenerative disorder with variable pathology arising from the complex interplay between one's genes and environment. Large‐scale genome‐wide association and related studies have identified many causal and contributing variants to early and late‐onset AD; yet the evolving polygenic risk of AD with time remains unknown. Nonhuman primate animal models like the common marmoset allow for the longitudinal assaying of disease progression; with a proportion of animals exhibiting AD‐like neurodegeneration as they naturally age. Here, we have analyzed genetic associations with plasma biomarker levels for the first time point in a cohort of laboratory marmosets of varying age.

**Method:**

Whole genome sequencing was carried out for a cohort of over a 100 largely wild‐type and a few *PSEN1* C410Y mutant carriers. Disease associated plasma biomarkers were measured for assessing the progression of neurodegeneration. The correlation structure underlying collected biomarkers and related phenotypes was calculated, separating for sex. Composite phenotypes were computed by extracting the principal component explaining the most variance for biomarkers of interest, and genetic associations assessed via tools implementing a linear mixed model for composite and individual phenotypes while adjusting for kinship. Permutation testing was done to identify a robust *p*‐value threshold for GWAS, and potential variant loci of interest were examined.

**Result:**

Females showed greater correlations compared to males between biomarkers specific to neurodegeneration, like total tau (tTau) and neurofilament light protein (NfL), in addition to the levels of the marker of astrocytic activation GFAP with the beta‐amyloid peptides containing 40 and 42 amino acids. A statistically significant positive correlation of NfL with age was observed only in males. Many significant and suggestive SNPs were found to be associated with the biomarkers under study in our genome‐wide scans, and their functional impacts are currently being investigated.

**Conclusion:**

Variation in AD‐relevant biomarkers in marmosets were not explained solely by age, suggesting a strong potential genetic contribution. Our work aims to leverage possible heritable associations with disease markers discovered in the marmosets to explain the progression of neurodegeneration, which could directly provide insights into human AD biology from a genetic point of view.

